# Molecular eyes: proteins that transform light into biological information

**DOI:** 10.1098/rsfs.2013.0005

**Published:** 2013-10-06

**Authors:** John T. M. Kennis, Tilo Mathes

**Affiliations:** Biophysics Group, Department of Physics and Astronomy, Faculty of Sciences, Vrije Universiteit, De Boelelaan 1081, 1081 HV Amsterdam, The Netherlands

**Keywords:** photoreceptor, flavin, proton-coupled electron transfer, spectroscopy

## Abstract

Most biological photoreceptors are protein/cofactor complexes that induce a physiological reaction upon absorption of a photon. Therefore, these proteins represent signal converters that translate light into biological information. Researchers use this property to stimulate and study various biochemical processes conveniently and non-invasively by the application of light, an approach known as optogenetics. Here, we summarize the recent experimental progress on the family of blue light receptors using FAD (BLUF) receptors. Several BLUF photoreceptors modulate second messenger levels and thus represent highly interesting tools for optogenetic application. In order to activate a coupled effector protein, the flavin-binding pocket of the BLUF domain undergoes a subtle rearrangement of the hydrogen network upon blue light absorption. The hydrogen bond switch is facilitated by the ultrafast light-induced proton-coupled electron transfer (PCET) between a tyrosine and the flavin in less than a nanosecond and remains stable on a long enough timescale for biochemical reactions to take place. The cyclic nature of the photoinduced reaction makes BLUF domains powerful model systems to study protein/cofactor interaction, protein-modulated PCET and novel mechanisms of biological signalling. The ultrafast nature of the photoconversion as well as the subtle structural rearrangement requires sophisticated spectroscopic and molecular biological methods to study and understand this highly intriguing signalling process.

## 1. Introduction

Light is not only one of the major foundations for the development of life on the Earth as we experience it today, but it also serves as an important sensory input not only for plants but also for the other phototrophic organisms. Phototrophic organisms need to carefully balance the exposure of their photosynthesis machinery to varying levels of light between optimal photosynthetic efficiency and protection from photoinduced damage by reactive oxygen generation. In addition, most single-cellular organisms such as bacteria and fungi, for example, depend on efficient photoprotection mechanisms in order to avoid or tackle harmful UV radiation, which may damage DNA or create reactive oxygen species. Most mammals and other higher non-plant organisms rely on light as an additional sensory input to forage for food and to avoid enemies or harmful situations, etc. For the perception of light in the UV/vis range, nature has developed sophisticated sensory machineries in all kingdoms of life. In higher animals, whole organs, eyes, have evolved [1], which add the experience of direction and distance of a light stimulus to the very basic photoreception machinery. Some microbes such as the unicellular alga *Chlamydomonas reinhardtii* evolved a sophisticated anisotropic shading mechanism that allows them to perceive the direction of light relative to their swimming direction [2]. The core compound of every eye however is a rather ‘simple’ photoreceptor molecule. These photoreceptors usually consist of small organic molecules that absorb light of a specific wavelength. Most of these pigments have been present already since the early days of life, and nature has developed sophisticated machineries to use them as the primary interface between light and biology. To build a functional photosensor, which can translate the ultrafast event of light absorption into biological information, these pigments are embedded into a protein matrix. In classical cellular receptors, which bind small molecules or more complex hormones, a structural change is triggered upon the binding of the messenger compound to the receptor. In most photoreceptors, the pigment cofactor is permanently bound and only upon absorption of a photon induces structural changes in the protein scaffold and triggers signal transduction subsequently.

**Figure 1.**
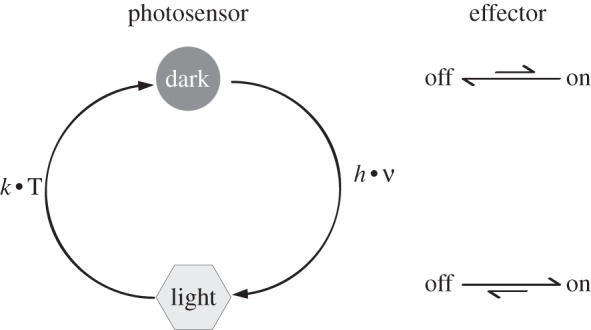
Most biological photoreceptors can be described as light-activated switches, which thermally recover to the dark-adapted state. Upon transition from a dark-adapted state to a light-adapted state, the photosensory part of the protein modulates the activity of the cognate effector component.

Because the primary photoreception event, absorption of a photon, leads to an electronic-excited state of the chromophore that may only live for picoseconds to nanoseconds or even less, a chemical or structural change has to be induced that persists for orders of magnitude longer. This is very crucial not to lose the just received information, because the structural rearrangement into the signalling form of the receptor and also subsequent biochemical processes usually occur on much longer timescales. In most cases, these so-called signalling states are metastable and recover thermally to the dark-adapted state. Thereby, most photoreceptors represent intrinsic cyclic reaction systems that may be investigated with reaction-induced difference spectroscopy. Because light is the actual trigger, the reaction may be induced with ultrafast temporal precision down to few femtoseconds with state-of-the-art pulsed laser sources. In contrast to substrate or chemically induced reactions using stopped flow technology, which usually have dead-times of a few hundreds of microseconds [3], femtosecond time-resolution may be achieved for laser-induced reactions. Skeletal motions of chromophore and protein components take place in the subpicosecond timescale [4] and may be resolved spectroscopically to obtain a complete picture of signal generation and propagation. It should be noted however that although in most cases the photoinduced transformation of the chromophore binding pocket in the photosensory part is usually a clear on/off response, whose effectivity is solely determined by its quantum efficiency, the biological effect mediated by more distal parts in the photoreceptor is, in many cases, more of a modulation of signalling rather than an on/off switch (figure 1). The photosensor therefore shifts the equilibrium between the biological on and off states of the effector.

The unique ability to manipulate biological processes by light using such photoreceptor proteins is the foundation of the recently emerging field of optogenetics [5]. Optogenetics has become a key technology in the past few years, because the actuator can be genetically encoded and its activation can be accomplished in a non-invasive way with the highest spatio-temporal precision. Photoreceptor proteins may be introduced functionally into virtually any cell type and various effects may be induced by the application of light. The so-far-used photoreceptors enabled researchers to stimulate neuronal cells using rhodopsin-based ion channels or pumps [6], manipulate second messenger levels [7–11] and regulate gene expression by the application of light [12,13]. Because light of all feasible wavelengths may be applied with high temporal and spatial precision, selective stimulation of certain cell types and even mapping of neuronal circuits has been accomplished [14]. Furthermore, cell-specific promoters may be used to selectively render a certain cell-type responsive to light. These photoreceptors may also be used as powerful components in synthetic biology to act as switches in engineered biological machines [15]. A deeper knowledge of the underlying molecular mechanisms of signal transduction is mandatory to rationally customize these photoreceptors for their specific purposes and applications.

In the following sections, we illustrate that the study of photoreceptor proteins is not only of interest to understand the molecular mechanisms of light perception and signal transduction but also to study fundamental reactions in chemistry and biology.

## 2. BLUF photoreceptors

**Figure 2.**
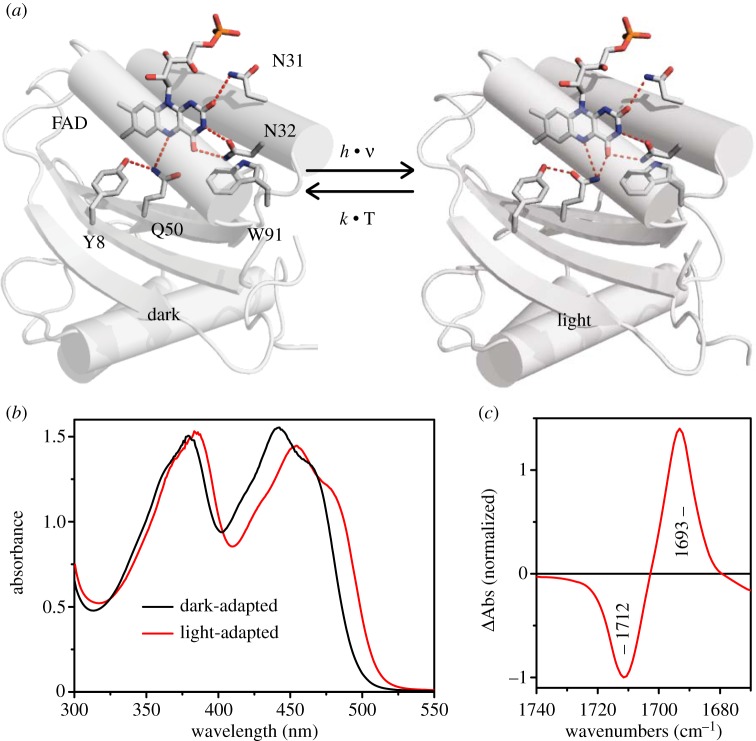
(*a*) BLUF domain of Slr1694 in dark- and light-adapted states illustrating the putative glutamine rotation mechanism. (*b*) The visible absorbance spectrum changes shifts by about 15 nm to the red upon illumination. (*c*) The light-minus-dark FT-IR difference spectrum shows the downshift of a carbonyl signature by approximately 20 cm^–1^ predominantly assigned to the hydrogen bonding to the C_4_=O of the flavin.

Here, we present the reader an overview of our recent experimental work on blue light receptors using FAD (BLUF) photoreceptors [16]. This photoreceptor family is found mainly in prokaryotes but also in eukaryotic single-cellular microbes. BLUF photoreceptors have first been shown to be responsible for photoprotective reactions such as phototaxis [17] but lately have also been found to regulate lifestyle decisions, for example biofilm formation and virulence [18–20]. BLUF photoreceptors are modular photoreceptors with an about 150 amino acid containing receptor domain—the hence named BLUF domain—forming a ferredoxin-like βαββαβ fold [21–27] (figure 2*a*). Many BLUF domains are part of larger proteins C-terminally connected to enzymes as effector domains, which are involved in second messenger synthesis or breakdown. The activity of these effectors is modulated by the BLUF domain in response to light conditions [10,11,28]. Other members of the BLUF family are fused to transcriptional effectors for light-dependent regulation of gene expression [29]. In addition, only the BLUF domain containing proteins with short C-terminal, mainly helical extensions, are frequently found. These proteins transmit signals by light-dependent protein/protein interaction [30–32]. In particular, the BLUF-regulated enzymes have drawn major attention as optogenetic tools that can be used in cell biology and neurobiology as light-induced actuators to study, for example, second messenger-related physiological responses [7–11,33]. Due to their modular architecture, BLUF domains can be interchanged between different photoreceptors [34] and may even be functionally fused to effectors not found in nature as observed for the modular LOV photoreceptors [13,35–40]. In all the above-mentioned signal transduction scenarios, the BLUF domain has to undergo structural rearrangements upon illumination, which modulate its interaction with the effector protein/domain. The BLUF domain binds flavins—FAD/FMN/RF [41]—non-covalently and uses their isoalloxazine moiety as a pigment to absorb blue light. Unlike other well-studied photoreceptors such as rhodopsin and phytochrome, flavin-binding photoreceptors contain a rigid, non-isomerizable cofactor and therefore challenge scientists with new ways of phototransformation of the pigment [42]. Flavin-containing photoreceptors belonging to the LOV and cryptochrome family undergo light-induced transformations similar to what is known from flavoenzyme intermediates such as covalent adduct formation or redox reactions, respectively [43]. The BLUF domains, by contrast, merely show a rearrangement of hydrogen bonds around the flavin cofactor after illumination. This results in a red shift of the BLUF domain-visible absorbance spectrum by 10–15 nm (figure 2*b*). The nature of this hydrogen bond network in both the dark- and the light-adapted state is still poorly understood and is discussed controversially. This is mainly due to differences in so-far-determined NMR and crystal structures of BLUF domains and their assignment to dark- and light-adapted states [21–24,27]. The commonly agreed on components of the light-activation mechanism in BLUF domains include two essential amino acids, a conserved glutamine and a conserved tyrosine (figure 2). Photoactivation is inhibited upon the removal of these amino acids [44–46]. Hydrogen bonding to C_4_=O of the flavin in the light-adapted state is indicated by a downshift of flavin carbonyl frequencies observed in light-minus-dark FT-IR difference spectra (figure 2*c*) [47]. The responsible hydrogen bond is most likely donated by the conserved glutamine residue, which may either rotate (figure 2) or, as indicated by theoretical calculations [48–52], tautomerize in the light-adapted state to facilitate the new hydrogen bond. The hydrogen bond rearrangement is initiated by a series of ultrafast reactions, including photoinduced proton-coupled electron transfer (PCET) within less than a nanosecond [53,54]. The hydrogen-bond-switched state, however, is stable for nine orders of magnitude longer and exclusively recovers thermally without any light-induced back reaction [55]. These subtle structural changes around the chromophore inside the protein core are transmitted to the surface of the BLUF domain and lead to activation of an associated effector protein or an internal effector domain. The molecular details of this process are so far not very well understood, because very little information is available on the structural differences in light- and dark-adapted state of full-length photoreceptors or BLUF/effector complexes [28]. A clear peripheral structural component of the signal transduction process is found only in a highly conserved methionine residue, which might change its conformation from a buried position that is close to the flavin, to a more distal position (or vice versa) along with a displacement of the β5 sheet [21,23]. The β5 sheet is apparently of central role, since mutations that affect its structural integrity also affect the equilibrium between signalling active and inactive states [32]. Upon removal of the methionine, BLUF domains become unable to activate downstream effectors although the hydrogen-bond-switched state is still formed [56]. Furthermore, one should consider that the inter/intramolecular signal transduction pathways might differ significantly within the BLUF family similar to what was previously observed for the similar modular LOV-domain photoreceptors [57].

Because of their unique photoactivation as outlined above, BLUF domains are interesting model objects to investigate protein-modulated, proton-coupled electron transfer, dynamic hydrogen networks in biological systems as well as new paradigms of light-mediated signal generation and control in biology. The latter is especially interesting for neuro- and cell biologists, who are interested in the customized variants of existing photoreceptor/effector systems with selected sensitivities and activities for their biological questions at hand. Therefore, it is necessary to fully understand the mechanisms of the natural photoreceptors. Sophisticated biophysical techniques and molecular biological tools as outlined below are necessary for this purpose.

## 3. Ultrafast structural responses in BLUF photoreceptors upon illumination

**Figure 3.**
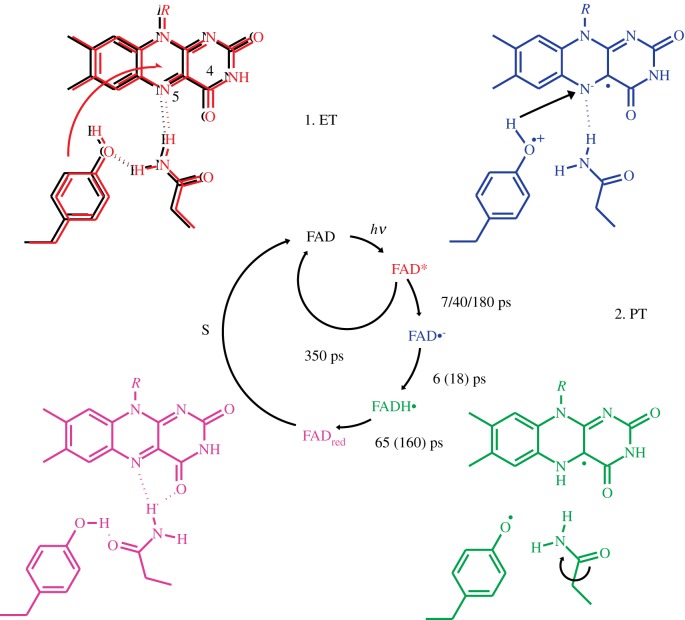
Photocycle of BLUF domains as observed by ultrafast vis/IR and fluorescence spectroscopy on Slr1694. Lifetimes printed in parentheses correspond to H/D isotope affected reaction rates observed in D_2_O.

Because the hydrogen bond switch occurs in less than a nanosecond, only ultrafast spectroscopic techniques such as pump/probe absorbance spectroscopy [58] or ultrafast time-resolved fluorescence spectroscopy [59] are able to address its molecular details. However, not only the choice of experimental techniques is crucial to obtain detailed insights but also the choice of the model system turned out to be critical. In early ultrafast studies on AppA, a light- and redox-dependent transcriptional regulator of photosynthetic gene expression in *Rhodobacter sphaeroides*, it was found by both absorbance and emission spectroscopy that the flavin in BLUF domain exhibits a complex multiexponential excited state decay after illumination of the dark-adapted state [60,61]. This behaviour was later confirmed to be a characteristic property of BLUF domains, in general, and is most likely due to conformational heterogeneity in the ground state of the photoreceptor [27,53,62,63]. Due to the broad range of excited state lifetimes, ranging from tens to thousands of picoseconds in AppA, only excited state and signalling state features were obtained by ultrafast spectroscopy. The phototaxis-related single BLUF domain protein Slr1694 from *Synechocystis* sp. PCC 6803 [56,64]—also referred to as SyPixD—in contrast features a much faster excited state decay than AppA and was able to provide the first and up to now still the most insight into the reaction intermediates during BLUF photoactivation [54]. Due to a strong inverted kinetic behaviour—some intermediates decay faster than they are formed from the excited state—so far it has been only possible to resolve the photocycle intermediates kinetically in Slr1694 (figure 3). Nevertheless, it remained necessary to apply sophisticated data analysis procedures to extract the true spectra of the reaction intermediates. Transient absorption and fluorescence can be experimentally determined by recording absorbance/intensity changes at a single wavelength. However, by recording transient spectra covering a complete wavelength region of the absorbance/intensity change, a data matrix is obtained, in which the spectral change is resolved in both wavelength and time. Such datasets allow the estimation of the number of relevant components by singular value decomposition and the application of global analysis procedures [65,66]. Global analysis can be used to fit the raw data using compartmental models. A useful first evaluation of the spectral evolution may be obtained by using a sequential model where the data are fitted by sequentially interconverting species with increasing lifetimes. Thereby, the obtained evolutionarily associated spectra do not necessarily represent the spectra of the true species of the reaction intermediates, but give a good overview on the temporal evolution of the absorbance/intensity changes. Using rational considerations backed up by kinetic properties observed in the raw data, experimental parameters or the knowledge of spectral properties of an intermediate even allows us to impose inverted kinetics and more or less complicated branching of the reaction on the dataset. Additional constraints may be introduced by simultaneous analysis of datasets recorded under different conditions, e.g. H_2_O- or D_2_O-buffered samples (see below). This so-called target analysis is able to extract the species-associated difference spectra, which ideally represent the true spectra of the corresponding molecular species.

By a combination of time-resolved fluorescence, ultrafast visible and IR absorption spectroscopy and global analysis the following photocycle was established for Slr1694 (figure 3) [53,54]. This combination of spectroscopic techniques sensitive to both the electronic properties of the flavin as well as structural dynamics of flavin and protein recorded with femtosecond time-resolution proved to be crucial to obtain the most complete description of BLUF photoactivation so far. After femtosecond excitation of the flavin, the heterogeneous-excited state is quenched by electron transfer from the nearby tyrosine with an average lifetime of 17 ps [67]. Although the role of tyrosine as the primary electron donor was already highly suggested from the study of site-direct mutants, the first experimental evidence was provided by ultrafast IR spectroscopy [53]. An aromatic signature of the phenolic side chain was kinetically resolved from the flavin signatures and clearly showed its involvement in the electron-transfer process. The resulting anionic flavin semiquinone is quickly protonated, most likely by the same tyrosine. The sequence of reactions was confirmed by H/D-isotope-dependent measurements, which showed that the excited state decay as observed by both visible absorption and more exclusively by fluorescence spectroscopy is H/D-isotope independent, whereas the subsequent reaction shows a kinetic isotope effect of 3 in D_2_O-buffered solution. The reaction is therefore classified as a sequential PCET process, where proton transfer follows electron transfer. The protonation of the flavin semiquinone may provide a trigger for the subsequent rotation of the glutamine amide, because the hydrogen bond between glutamine and N5 is broken in the process. The neutral flavin semiquinone/tyrosine radical pair recombines within 60 ps by another H/D-dependent PCET reaction. The flavin is again present in the oxidized form, but the visible and IR spectra are shifted and characterize the hydrogen-bond-switched state. The IR spectrum of the hydrogen-bond-switched state after 1 ns differs from steady-state FT-IR difference spectra and indicates further structural relaxation processes. Indeed, slower processes take place on the microsecond and millisecond timescale possibly involving subtle protonation changes at a conserved aspartate [68] and changes in the oligomeric state of the protein, respectively [31,69]. These changes are most likely associated with signal propagation to peripheral regions of the BLUF domain, which ultimately lead to the activation of the corresponding effector.

Modifications in the flavin-binding pocket by site-directed mutagenesis showed that photoactivation of BLUF photoreceptors relies on very specific hydrogen bond networks and very specific chemical reactivities. Removal of the hydroxyl group of the conserved tyrosine by a mutation to phenylalanine abolished the formation of the red-shifted state [45], probably due to the removal the proton donor for formation of the neutral flavin semiquinone intermediate and the altered midpoint potential of the donor. Moreover, an important hydrogen bond interaction with the conserved glutamine is removed in such a mutated protein. Accordingly, the removal of the conserved glutamine prevents signalling state formation [25], although a neutral flavin semiquinone is formed in the Slr1694 Q50A mutant that lives for a few nanoseconds (T. Mathes & R. Fudim 2011, unpublished data). By replacement of the phenol side chain by an indole (Y8W), which, in principle, should provide a similar chemical basis to both donate electrons and protons to the flavin-like tyrosine, we also observed the formation of a mixture of several flavin radical pair species, which however also did not yield the red-shifted signalling state [70,71]. These studies showed that not only light-induced PCET is required for BLUF photoactivation but also defined interactions between protein and flavin are necessary for the hydrogen bond switch to be facilitated. In the following, we will describe how the hydrogen bond network determines the nature of PCET in BLUF domains and its implications on the molecular details of dark- and light-adapted states in BLUF photoreceptors.

## 4. Proton-coupled electron transfer in BLUF domains: hydrogen bond tuning

Proton-coupled electron transfer involving a conserved tyrosine is a key reaction in solar energy conversion in the oxygen-evolving complex of photosystem II [72]. Because its purpose is to facilitate charge separation, the reaction is bidirectional, where electrons and protons are going separate ways. Its mechanistic details, especially the sequence of electron- and proton-transfer events, are still under considerable discussion. In protein environments, these properties are determined by discrete dipolar and hydrogen bond interactions, and structural restraints that have been evolutionarily optimized. Hydrogen bond networks involved in dynamic processes are generally difficult to investigate closely by crystallography and NMR spectroscopy because protons are very difficult to observe in X-ray diffraction experiments, and both crystallography and NMR spectroscopy do not offer the required time-resolution. Additionally, the study of PCET in a photosystem is difficult due to the large size of the protein complex, and most mechanistic studies are therefore performed on artificial model systems. BLUF domains may serve as a naturally occurring, suitable model system for protein-modulated PCET, because the hydrogen network can be reversibly perturbed by light and electronic, and structural dynamics can be monitored by vis, fluorescence and IR spectroscopy with femtosecond time-resolution.

**Figure 4.**
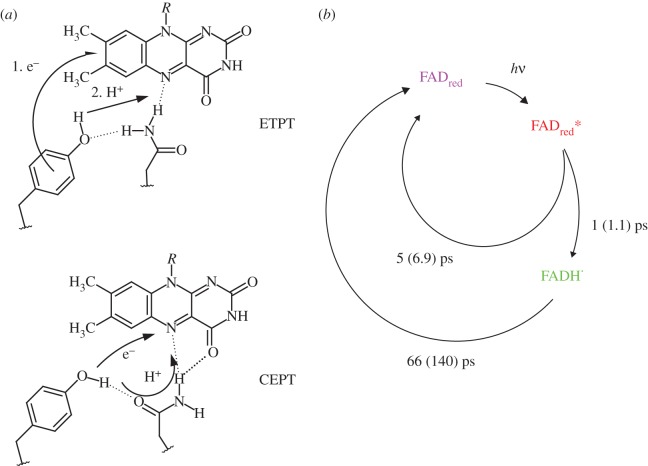
(*a*) The hydrogen bond network between tyrosine, glutamine and the flavin determines light-induced proton-coupled electron transfer. In contrast to the sequential ETPT reaction in the dark-adapted photocycle, the neutral semiquinone intermediate (*b*, green) in the light-adapted state is formed via a concerted ETPT (CEPT) reaction in about 1 ps. Lifetimes printed in parentheses correspond to the reaction rates observed in D_2_O.

In BLUF domains, we observe light-induced, proton-coupled electron transfer in a unidirectional manner from tyrosine to flavin [54]. The sequence of electron transfer proton transfer (ETPT) events may be altered by subtle modifications of the hydrogen bond network. In the dark-adapted state configuration, the reaction is strictly sequential where electron transfer takes place prior to proton transfer. Once electron transfer is accomplished within 17 ps, proton transfer follows in a few picoseconds to yield the neutral flavin semiquinone intermediate. Due to its long lifetime and its photoirreversibility, the hydrogen-bond-switched state can be prepared quantitatively by moderate background illumination [55]. In the light-adapted state, where the hydrogen bond network between flavin and the protein is altered, the same neutral flavin semiquinone is formed upon laser excitation according to ultrafast vis- and IR spectroscopy on the light-adapted state [55,73]. The reaction is now highly concerted (figure 4). This switched reactivity is not only interesting because of its molecular implications on the nature of the light- and dark-adapted states. It also shows that BLUF domains are potent model systems for protein-modulated PCET, because not only PCET can be studied with femtosecond resolution but also its reactivity can be conveniently switched by application of light.

Because PCET appears to be accomplished in a highly concerted manner, certain molecular prerequisites must be met. The short lifetime of the excited state and the fast formation of the neutral flavin semiquinone generally suggests that electron and proton donor must be in a tight configuration that is optimized for both electron and proton transfer. This is also already suggested by the lesser heterogeneity in the excited state when compared with the dark reaction [55,62,73]. Electron transfer is expected to occur directly from tyrosine to flavin due to the short distance of about 4–5 Å (edge to edge) and no apparent intermediate electron acceptors. Proton transfer, however, may be assisted by the hydrogen bond network around the flavin. Because N5 of the flavin is expected to be already hydrogen bonded in the light-adapted state as previously observed by ultrafast IR spectroscopy [53], we consider it unlikely that the proton or a hydrogen atom will be directly transferred from the tyrosine. In addition, the lack of a strong kinetic H/D-isotope effect suggests that the binding pocket is preconfigured for proton transfer to the flavin, which may only be facilitated by a strongly coordinating group. From FT-IR and NMR studies, we also know that tyrosine and glutamine form an unusually strong hydrogen bond in the light-adapted state [74,75], which we believe may only be realized by the coordination of the glutamine carbonyl group by the tyrosine hydroxyl group as displayed in figure 4. Therefore, we expect proton transfer to be mediated by the glutamine as a proton translocator in a Grotthus-like mechanism. A tautomeric form may occur in the process but should quickly revert to the thermodynamically more favourable amide form.

## 5. Photoinduced electron transfer in BLUF domains: redox tuning

As described above, the hydrogen bond network between flavin and protein determines the nature of the photoinduced PCET process. We also found the redox potential of both flavin and tyrosine plays a critical role for PCET. Additionally, it also affects the signalling process in BLUF domains.

Of all the so-far-investigated BLUF domains, Slr1694 (SyPixD) and Tll0078 (TePixD) show the fastest excited state electron-transfer reaction from tyrosine to flavin. The molecular basis for this behaviour was unclear at first, because straightforward factors such as distance and orientation as determined by X-ray crystallography and NMR were either suggesting an opposite trend or were hard to describe exactly with the quality of the structural information at hand. Naturally, the redox potentials of flavin and tyrosine should give information on the thermodynamic properties of the electron-transfer reaction. Although the redox potential of the flavin for the two electron reduction was determined for AppA and mutations thereof [76], this is not directly possible for the one electron reduction of the flavin. In wild-type (WT) BLUF domains, a single reduction is experimentally not accessible, because the product is directly fed into the photocycle. In addition, the redox potential of the tyrosine is not accessible in this manner. Additionally, the differences in redox potential might be small and difficult to discriminate. Therefore, we were looking for alternative ways to tune the redox potential of both flavin and tyrosine in Slr1694 and compare the ultrafast electron-transfer reaction with WT and other BLUF domains.

As described above, the reactivity of PCET in BLUF domains can be switched by application of light and valuable information can be extracted from such experiments. This feature is very powerful, because the same protein can be used to study subtle influences on reactivities, and the switch is facilitated rather non-invasively and moreover conveniently. Classically, researchers introduce site-directed mutations at sites of interest to change the reactivity of a given protein. In many cases, the results of such studies have to be treated with great care, because one cannot generally assume that only the local environment at the site of the mutation is altered. Additionally, the desired mutation may not fold or yield a functional protein. As an alternative to site-directed mutation, which is limited to the 20 canonical amino acids in nature, one may introduce chemical analogues for the cofactor or amino acids. For this purpose, it may be necessary to genomically modify the given expression system in order to disrupt endogenous synthesis of the cofactor or the amino acid. For *Escherichia coli*, such molecular biological procedures for genome engineering are well established and use homologous DNA recombination—also known as recombineering [77], facilitated by the overexpression of recombinases derived from bacterial viruses, so-called phages.

**Figure 5.**
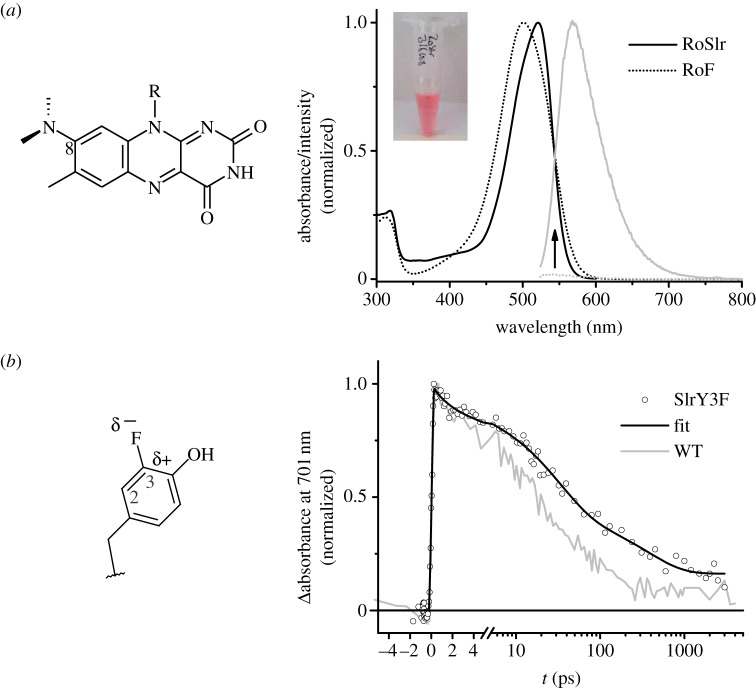
Chemically modified BLUF domains. Roseoflavin (RoF) was reconstituted into the BLUF domain (RoSlr) *in vivo* using a genomically engineered *E. coli* expression strain, which is devoid of flavin biosynthesis and capable of taking up riboflavin from the medium. (*a*) Absorbance and emission properties of the isolated protein are shifted to the red accordingly. Additionally, the fluorescence of roseoflavin increases about 60-fold upon binding to the protein. (*b*) The protein may also be chemically modified by introduction of non-natural amino acids such as fluorotyrosine using a tyrosine auxotrophic expression strain. The time-resolved absorbance change at 701 nm is characteristic for excited state decay of flavins, which is mainly determined by electron transfer from a conserved tyrosine. Compared with WT, the ET reaction is significantly slowed down. (Online version in colour.)

Flavin analogues have been successfully used as reactivity probes to study flavoenzyme mechanisms *in vitro* [78,79] and also have been applied recently to flavin-based photoreceptor proteins [80–85]. These analogues are usually introduced into a flavoprotein after the natural flavin cofactor has been released by (partial) unfolding and removed [86]. This procedure is possible for many flavoproteins, but not all proteins can be successfully stripped off their natural cofactor and refolded in the presence of the flavin analogue. Roseoflavin (figure 5*a*), a naturally occurring flavin analogue with antibiotic properties [87], has been shown to inhibit or alter the action spectrum of photoinduced physiological responses in microbes that are known to use LOV and BLUF photoreceptors [88,89]. Its redox potential is only by approximately 14 mV lower than that of riboflavin [90] and its approximately 50 nm red-shifted absorbance made it appealing to use it as a functional flavin analogue that can shift the action spectrum of a flavo-photoreceptor. In contrast to retinal and tetrapyrrole pigments, the flavin absorption can be shifted only in a very narrow regime through modifications of the binding pocket. First attempts to reconstitute Slr1694 with roseoflavin were unsuccessful using the classical approach mentioned above. However, we were able to reconstitute Slr1694 *in vivo* during heterologous expression in *E. coli* [85]. This approach is expected to be more gentle and efficient, because the flavin analogue is provided in the native folding environment of the cell. To facilitate quantitative incorporation of roseoflavin into Slr1694, we genomically modified an *E. coli* expression strain to produce a riboflavin transport protein from *Corynebacterium glutamicum. Escherichia coli* naturally has no need for a riboflavin uptake system because it is able to produce riboflavin itself. Because the endogenously produced riboflavin competes successfully with the externally supplied roseoflavin we additionally disrupted riboflavin biosynthesis by deletion of the riboflavin synthase *ribC* in this strain by genomic engineering. The flavin analogue in such a reconstitution experiment is then conveniently provided in the culture medium in its riboflavin form and converted into its corresponding FMN or FAD form after uptake inside the cell by the endogenous flavokinase.

With this strain, we were able to fully reconstitute Slr1694 with roseoflavin for spectroscopic studies [84,85]. The hence called RoSlr protein was studied by absorbance and fluorescence spectroscopy (figure 5*a*). To our surprise, the protein did not undergo any light-induced spectral changes in the visible region. This was quite unexpected, because the redox potential of roseoflavin is rather similar to riboflavin and should allow for efficient electron transfer and therefore initiation of photoactivation. Compared with riboflavin, roseoflavin is rather weakly fluorescent indicating a fast excited state decay [91]. Interestingly, the fluorescence quantum yield of roseoflavin emission was increased  approximately 60-fold upon binding to the BLUF domain [84,85]. Using time-resolved fluorescence spectroscopy, Penzkofer and co-workers were able to determine also the excited state lifetime of about 17 ps, which turned out to be significantly longer than for roseoflavin in solution of about 0.9 ps. The short-excited state lifetime is due to the presence of the dimethylamino group at the 8 position of the isoalloxazine ring. This functional group enables roseoflavin to undergo internal charge transfer (ICT) from the dimethylamino group to the pyrimidine part of the isoalloxazine ring. The dynamics of the (ICT) process are apparently influenced by the viscosity of the environment. At low temperatures as well as in the protein, the fluorescence is increased. This strongly suggests that the ICT involves structural rearrangements. A similar temperature-dependent behaviour has been observed by ENDOR spectroscopy for the rotation of the 8-methyl group of FMN in LOV-domains, which has been correlated with its interaction with the protein environment [80]. A likely explanation for the increased fluorescence of roseoflavin in the protein may be the presence of a molecular rotor formed by the dimethylamino group. ICT processes involving twisting of molecular rotors are classified as twisted internal charge transfer (TICT). Due to their high susceptibility to changes in local viscosity, fluorescent dyes with molecular rotors are used as probes for intermolecular interactions and local viscosity changes [92]. Because roseoflavin is slightly larger than riboflavin the rotation of the dimethylamino group may be restricted in the BLUF domain and TICT is rendered less efficient. Although the excited state of roseoflavin was thereby extended into a regime similar to the excited state lifetime of riboflavin in BLUF domains, still no initiation of the photocycle was observed. Therefore, one should also consider that the tyrosine/isoalloxazine orientation might have been changed in disfavour of electron transfer due to the additional, more bulky dimethylamino group of roseoflavin. In a recent theoretical work, Merz and co-workers found that the fluorescence-quenching behaviour in the protein would be, indeed, supported by a TICT mechanism; however, the loss of photoactivation may rather be due to a missing low-lying conical intersection between the locally excited state energy surface and the tyrosine/flavin charge transfer state surface [93].

Instead of using flavin analogues, the redox potential of the flavin/tyrosine redox pair may also be discretely modified by the flavin-binding pocket. We recently showed that the rate of electron transfer significantly depends on the redox potential of both flavin and tyrosine [67]. To change the redox potential of the flavin, we used a standard site-directed mutagenesis approach to introduce a positive charge close to heteroatom-rich pyrimidine part of the flavin. Asparagine at position 31 was replaced by histidine and arginine. These particular amino acids are present in other members of the BLUF family and are therefore expected to be less disturbing than the introduction of an amino acid that does not occur at this position in nature. Because no functional substitution with a member of the naturally occurring canonical amino acids is available for the tyrosine reaction partner (see above), we chose to introduce fluorinated tyrosine analogues into the protein (figure 5*b*). The substitution of hydrogen by fluorine atoms in organic compounds is considered to be isosteric, but, of course, the polarity of the former C–H bond becomes inverted. Depending on the number of fluorine substituents on the phenol ring, the redox potential and the acidity of the hydroxyl group can be affected [94]. To accomplish a quantitative incorporation of such slightly modified unnatural amino acid analogues, the endogenous tyrosine production has to be inhibited, because the natural tyrosine would compete successfully with the analogue. This may either be accomplished using selective inhibitors [95] or by genetic modification of the expression host [67]. As long as the corresponding tyrosyl tRNA-synthetase recognizes the analogue, a global substition of the natural amino acid by the analogue is achieved. Because on the ultrafast timescale, only the immediate surrounding of the flavin may be generally considered relevant, the incorporation of fluorotyrosine in the periphery of the protein may be neglected for the here investigated ultrafast PCET processes. A site-specific replacement however may be accomplished if orthogonal tRNA/amino-acyl-tRNA-synthetase combinations are provided in the cell. This approach uses the replacement of the codon of the natural amino acid by the so-called amber stop codon, whose translational stop function may be surpressed if a cognate amino-acyl-tRNA is present [96,97]. Additionally, a customized amino-acyl-tRNA-synthetase selective for the corresponding amino acid analogue is required. The customization for selectivity however becomes increasingly difficult the more sterically and chemically similar analogue and natural amino acids become [98]. A site-specific labelling with the sterically very similar fluorotyrosine used here may therefore be only acomplished using cell-free expression sytems, where the corresponding orthogonal tRNAs are supplied preloaded with the chemical analogue [99]. This approach however easily becomes experimentally challenging regarding protein yield and the complex preparation of the amino-acyl-tRNAs. Similar to the approach for incorporation of flavin analogues *in vivo* as described above, we genomically engineered an *E. coli* expression strain for this purpose by deletion of the gene for the chorismate mutase/prephenate dehydratase (*tyrA*). The chemically synthesized tyrosine analogue was provided in the culture medium during the expression of the protein and was successfully incorporated into the protein.

**Figure 6.**
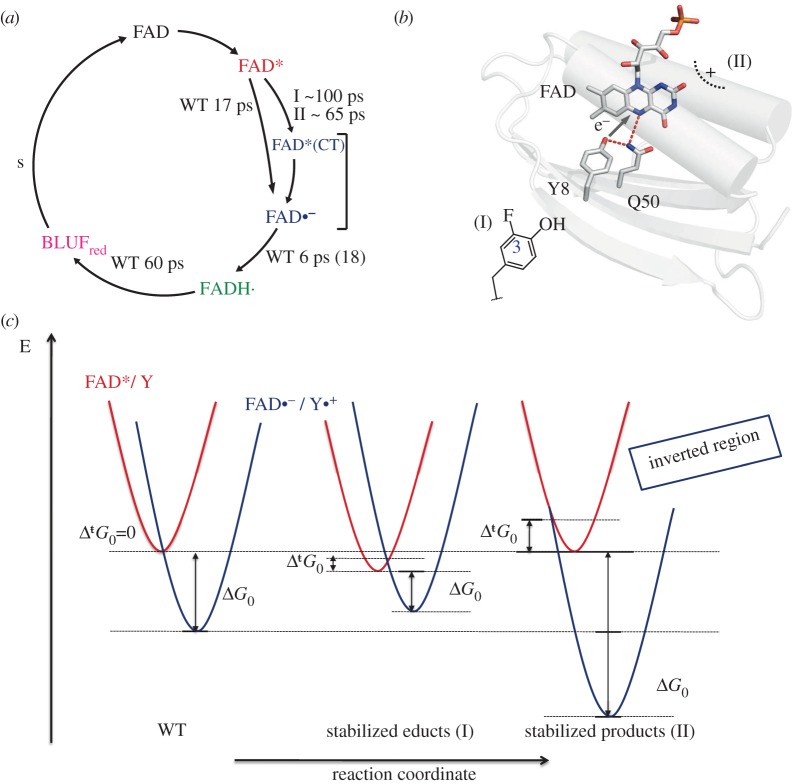
(*a*) Photocycle of BLUF domains as observed by ultrafast spectroscopy on redox-modulated Slr1694 proteins. Lifetimes printed in parentheses correspond to the reaction observed in D_2_O. (*b*) Photoinduced electron transfer from flavin to tyrosine in BLUF domains is significantly influenced by changes in the redox potential of tyrosine (I) and flavin (II). The slowed down electron transfer allowed us to observe an excited state charge transfer state (FAD*CT (*a*)), which cannot be resolved in WT. Electron transfer is highly optimized and almost activation barrierless in Slr1694 WT (*c*, left) and is significantly slowed down by either an altered tyrosine redox potential which disfavours PET (*c*, middle) or a PET favouring redox potential of the flavin redox partner (*c*, right).

Although the actual redox potential of flavin and tyrosine in these modified proteins may not be determined easily, several trends may be estimated. In WT, the redox potential for the single electron reduction of the excited flavin in its singlet state (FAD/^1^FAD*•–) is at around 1050 mV (NHE) [100], whereas the redox potential of the tyrosine (Tyr–O•/Tyr–O–) is expected at around 650 mV (NHE) [94]. The positive charge close to the flavin in the N31 mutants is expected to elevate the flavin redox potential. Therefore, the reduced form should be stabilized and the free reaction energy *ΔG*_0_ should become larger than in WT. In the fluorinated sample, the redox potential of the tyrosine is elevated in contrast, which then leads to a stabilization of the reduced tyrosine. Accordingly, the free reaction energy should become lower than in WT. Time-resolved spectroscopic experiments on these redox-modulated BLUF proteins showed that not only redox potentials that thermodynamically favour the educt states—fluorotyrosine proteins (figure 6*b*I)—but also redox potentials that favour the product states—N31 mutants (figure 6*b*II)—render electron transfer in BLUF domains less efficient than in WT (figure 5*b*). According to Marcus’ theory, this strongly suggests that electron transfer in Slr1694 is highly optimized and on the top of the Marcus’ curve (figure 6*c*) [101,102]. Slight variations of the redox potentials therefore either lower the free reaction energy or push the reaction into the inverted region (figure 6*c*), under the assumption that the reorganization energy is not affected in the modified proteins.

Redox modulation of the photodynamics of Slr1694 also turned out to be a perfect tool to investigate the electronic dynamics in the photocycle more closely. The slowed down electron-transfer rate provided us with an opportunity to catch a glimpse of a previously undescribed photocycle intermediate. In WT, the species-associated difference spectrum for the primary photoproduct perfectly describes an anionic flavin semiquinone with a significant CT character [54,67]. In both flavin and tyrosine redox-modulated proteins, we observed a similar intermediate, which additionally contained a stimulated emission feature, which indicates that an excited state intermediate is formed. The red-shifted nature of the SE in this intermediate is characteristic for an excited state flavin species with significant charge transfer from a nearby tyrosine [103]. This suggested that after excitation of the flavin, significant charge redistribution occurs from tyrosine to the flavin prior to full electron transfer. The FAD*/Y CT state is not visible in WT due to the fast electron-transfer rate and became partly observable in the redox-modulated proteins (figure 6*a*).

## 6. BLUF signalling

Interestingly, not only the primary photochemistry is significantly affected by the redox modulation of the primary ET process. The quantum yield of the hydrogen-bond-switched state is also influenced by the modulated electron-transfer rates as described above, and the observed correlation is in line with observations on other WT BLUF domains [67]. Apparently, a high electron-transfer rate correlates with high quantum efficiency for the hydrogen-bond-switched state. This red-shifted state, which is formed already after less than a nanosecond, is however not necessarily the final biological signalling state. Its formation is nevertheless most definitely a prerequisite for efficient activation of a downstream effector. Therefore, we believe that the redox potentials of flavin and tyrosine are evolutionarily fine-tuned in order to yield the desired optimal sensitivity for the corresponding biological context. Additionally, BLUF domains might use another mechanism in order to modulate the signalling quantum yield. The slower electron-transfer rate in AppA also allows for a competing electron transfer from a nearby tryptophan residue [104]. By electron transfer from the indole side chain, the formation of the hydrogen-bond-switched state is prohibited, because the flavin/tryptophan radical pair recombines back to the dark-adapted state. Accordingly, removal of the tryptophan increases the quantum yield of the red-shifted state significantly [105]. Probably, due to the larger tryptophan/flavin distance in other BLUF domains or due to faster electron-transfer rates, this mechanism has so far been only demonstrated for AppA. Furthermore, this tryptophan residue is not strictly conserved among the BLUF family.

Of course, the major interest in signal transduction in BLUF domains is the communication between the receptor and effector components. Studying this process is highly complicated due to the fact that not many well-defined BLUF receptor/effector complexes are available for *in vitro* study so far. Moreover, light-activated forms of these proteins have so far been inaccessible by crystallography and spectroscopic data such as infrared spectra are difficult to interpret precisely. Although light-minus-dark steady-state difference FT-IR spectroscopy is, in principle, able to elucidate the final structural differences between dark- and light-adapted states and can help to identify regions of interest in full-length photoreceptors [106], molecular interpretation is often elusive, because IR spectra are generally highly crowded. A clear assignment may be obtained by systematic isotope labelling of cofactor and/or selected amino acids of the protein, which is accessible via the above described genomically modified expression systems. One should note however that due to the almost identical sterical and chemical nature of the labelled and unlabelled amino acids, no site-specific delivery but only an amino-acid-type-specific delivery is likely possible in this way. This may be largely neglected on the ultrafast timescale but has to be clearly taken into account in the interpretation of steady-state IR spectra. As mentioned above, the only other option is using cell-free expression systems, which are provided with orthogonal tRNAs preloaded selectively with isotope labelled amino acids. Additionally, the structural propagation of the signal within the protein, which takes place during the transition between these two states and typically occurs in the nanosecond to millisecond time regime, is of high interest and may also help to describe the processes on a molecular level. These time-domains however are experimentally difficult to access by traditional vibrational spectroscopic methods. Ultrafast transient absorption spectroscopy is usually experimentally feasible only up to few nanoseconds and time-resolved FT-IR spectroscopy (Step-Scan) is difficult to perform for slowly relaxing systems such as the BLUF domains [68]. Therefore, the development of dispersive infrared spectrometers in combination with enhanced transient absorption setups using pairs of electronically synchronized Ti : sapphire laser systems is highly desirable to be able to describe this obvious gap in the structural dynamics of BLUF photoactivation and to be able to use wild-type proteins under natural conditions.

## 7. Perspective

BLUF photoreceptors are valuable model systems to probe the dynamic interaction of protein and cofactor. This knowledge is not only relevant for our understanding of BLUF photoreception but can be transferred to a more general (bio-)chemical context. From the study of BLUF proteins, we can learn how the protein environment determines fundamental (bio-)chemical reactions and how structural transitions are propagated within biomolecules. These processes can be monitored by high-end spectroscopic techniques sensitive to electronic and structural changes with temporal precision down to a few femtoseconds. In combination with state-of-the-art experimental techniques in molecular biology that expand our possibilities beyond the natural repertoire of amino acids and cofactors, we are able to successfully integrate our knowledge in chemistry, physics and biology. A deep understanding of the BLUF photoactivation mechanisms will finally enable us to rationally customize and design novel tools for optogenetics and synthetic biology with optimized activities and sensitivities for their corresponding application.
